# Objective assessment of surgeon kinematics during simulated laparoscopic surgery: a preliminary evaluation of the effect of high body mass index models

**DOI:** 10.1007/s11548-021-02455-5

**Published:** 2021-07-24

**Authors:** Ryan Sers, Steph Forrester, Massimiliano Zecca, Stephen Ward, Esther Moss

**Affiliations:** 1 Wolfson School of Mechanical, Electrical and Manufacturing Engineering, Loughborough University, Loughborough, UK; 2 Leicester Cancer Research Centre, University of Leicester, University Road, Leicester, LE1 7RH UK

**Keywords:** Laparoscopic surgery, Obesity, IMU, Kinematics, Workload

## Abstract

**Purpose:**

Laparoscopy is used in many surgical specialties. Subjective reports have suggested that performing laparoscopic surgery in patients with a high body mass index (BMI) is leading to increased prevalence of musculoskeletal symptoms in surgeons. The aim of this study was to objectively quantify the impact on surgeon upper body kinematics and dynamic workload when performing simulated laparoscopy at different BMI levels.

**Methods:**

Upper body kinematics and dynamic workload of novice, intermediate and expert surgeons were calculated based on measurements from inertial measurement units positioned on upper body segments. Varying thicknesses of foam were used to simulate patient BMIs of 20, 30, 40 and 50 kg/m^2^ during laparoscopic training.

**Results:**

Significant increases in the jerkiness, angular speed and cumulative displacement of the head, torso and upper arms were found within all experience groups when subject to the 40 and 50 kg/m^2^ models. Novice surgeons were found to have less controlled kinematics and larger dynamic workloads compared to the more experienced surgeons.

**Conclusions:**

Our findings indicate that performing laparoscopic surgery on a high BMI model worsens upper body motion efficiency and efficacy, and increases dynamic workload, producing conditions that are more physically demanding when compared to operating on a 20 kg/m^2^ model. These findings also suggest that the head, torso, and upper arm segments are especially affected by high BMI models and therefore exposure to patients with high BMIs may increase the risk of musculoskeletal injury when performing laparoscopic surgery.

## Introduction

Obesity has become one of the most prominent global health risks, with an incidence at an all-time high [1]. Body mass index (BMI), used as an indicator of body composition throughout healthcare, subdivides obesity into three categories: Class 1, BMI of 30 kg/m^2^ to < 35 kg/m^2^, Class 2, BMI of 35 kg/m^2^ to < 40 kg/m^2^ and Class 3, BMI of ≥ 40 kg/m^2^ often termed as ‘severe’ [1]. Obesity levels in England have increased from 15% of the adult population in 1993 to 27% in 2015 [2], with current projections of over 4 million adults with severe obesity by 2035 across England, Scotland and Wales [3].

Obesity is reported to be the second largest preventable cause of cancer and is linked to many malignancies, such as endometrial cancer [4]. Minimally invasive surgery (MIS), and specifically laparoscopic surgery (LS), is a primary method of treatment for many obesity-related cancers as the technique has a significantly better peri-operative morbidity/mortality rate compared to open surgery [5].

Laparoscopic surgery is associated with a significantly greater risk of work-related musculoskeletal disorders (MSDs) in surgeons, compared to open or robotic-assisted surgery [6]. The neck, back, shoulders, and knees are the most commonly reported symptom areas [7]. MSDs have a complex multifactorial aetiology; however, in surgeons, the incidence of such disorders is most widely attributed to the physical demands associated with LS including extreme operating postures leading to high muscular loading around the neck and trunk combined with awkward and repetitive upper arm movements, both experienced over extended periods of time [6, 7]. Additional contributing factors to MSDs include a higher number of surgeries performed in a given time period and patients with obesity [8]. While surgeons have anecdotally associated procedures on patients with high BMIs with increased workload, little objective research exists into understanding whether this statement has validity.

Recent studies have assessed the differences in surgeon muscle activity and kinematics when performing LS at BMIs < 30 kg/m^2^ (non-obese) and between 30 and 35 kg/m^2^ (Class 1) [9, 10]. Specifically, the first study assessed muscular stress of surgeons performing live LS by measuring muscle activity using electromyography finding no significant differences between BMI levels for all upper body muscles [9]. The second study measured the muscle activity of the back and the motion of the upper body using inertial measurement units (IMUs) fixed on the waist, torso, shoulders and upper arms when completing simulated LS and found significant differences (*p* < 0.05) in torso angular motion between BMI levels [10]. However, given the growing prevalence of severe obesity, studies investigating the effects of BMIs ≥ 40 kg/m^2^ on surgeon kinematics are needed.

The purpose of this study was to assess the impact of different levels of simulated patient BMI on surgeon upper body kinematics and the implications on the subsequent dynamic workload when performing LS. In addition, surgeons of varying experience levels were assessed to investigate the severity of BMI on MIS trainees (novices), early career surgeons (intermediates) and consultants (experts). To assess surgeon upper body kinematics and dynamic workload, two groups of metrics were considered. Firstly, the efficiency and efficacy of upper body kinematics were evaluated through mean linear jerk [11], mean angular speed and the cumulative displacement of body segments [12]. Secondly, global indicators of work done by the surgeon included the cumulative displacement of body segments together with the time required to complete the task [13]. The authors hypothesized that increased BMI would degrade surgeon kinematics and increase their dynamic workloads, and that the deterioration in these factors would be greatest for less experienced surgeons.

## Materials and methods

### Participants and ethics

This study was conducted at Loughborough University, UK. Ethical approval was granted by the University Ethics Approvals Sub-Committee and all participants provided voluntary informed consent prior to testing. The participant cohort consisted of three senior gynecological surgeons (10 + years of LS experience, > 100 procedures per year, referred to as ‘expert’ participants), three senior gynecological trainees (4—6 years’ LS experience, > 50 procedures per year, referred to as ‘intermediate’ participants) and four final year medical students (no live LS experience, referred to as ‘novices’) (6 males and 4 females, with heights: 168–188 cm, and masses: 59–96 kg). All participants had previous exposure to simulated environments, and all were right hand dominant.

### Instrumentation and equipment

**Fig. 1:**
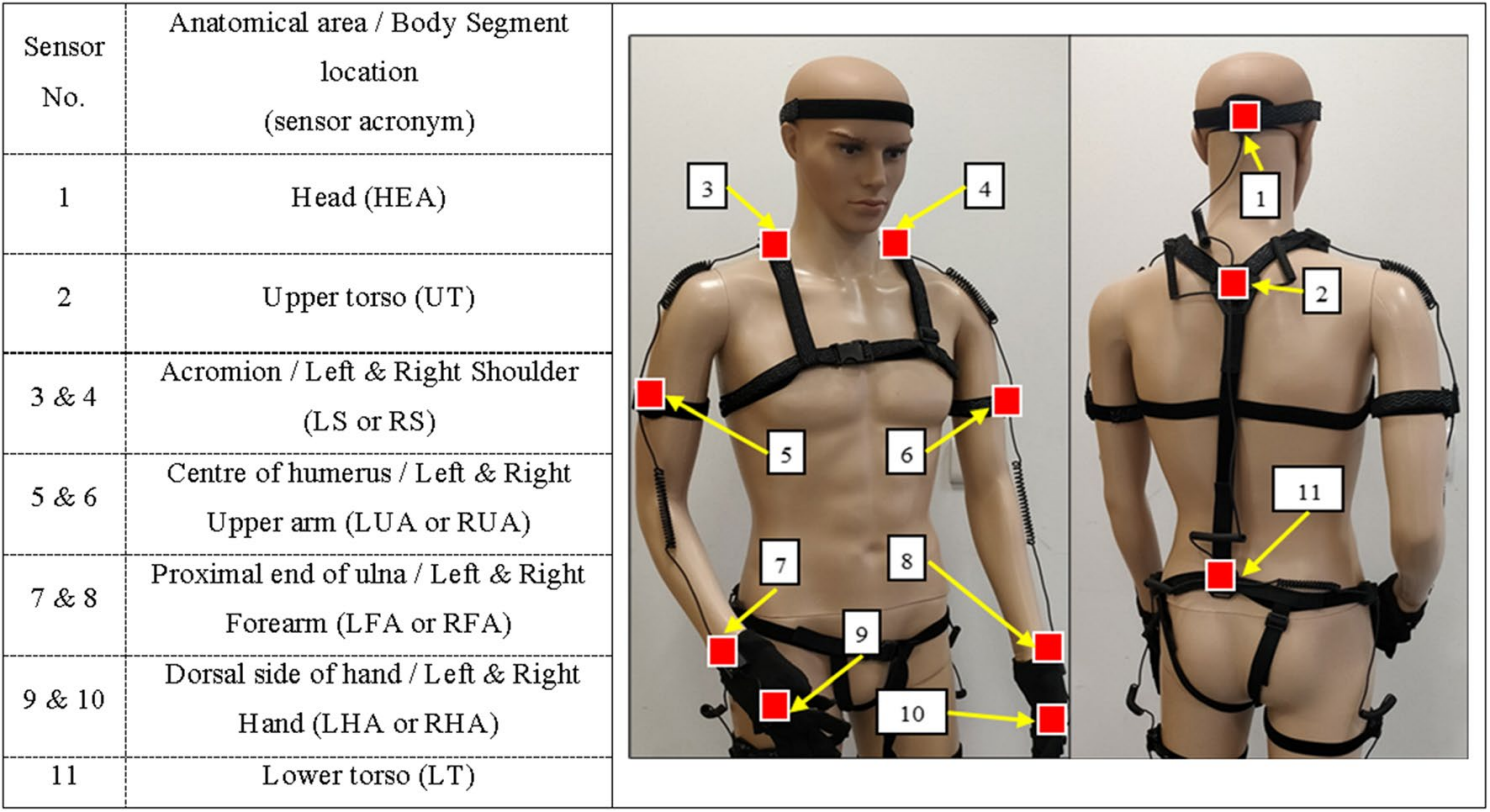
The IMU positions of the motion capture system highlighted with squares

To capture participant kinematics, the Perceptron Neuron inertial motion capture (Mo-Cap) system was used configured in the 18-neuron setup (NOITOM Ltd, China). The Mo-Cap system was setup in this mode despite the use of only 11 upper body sensors because the 18-neuron configuration has been previously validated for motion analysis (Fig. 1) [14]. The setup comprised of 11 inertial measurement units (IMUs); each IMU (size: 12.5 × 13.1 × 4.3 mm, mass: 1.2 g) consisted of a 3-axis accelerometer (± 16 g) and 3-axis gyroscope (± 2000 dps). The IMUs were connected in series to a wireless hub, which transferred the data from the suit’s sensors to a dedicated PC (at 120 Hz). Participant anthropometrics were measured using an Xbox Kinect 2 (Xbox, Microsoft, Redmond, WA, USA) and used to calibrate the system following its recommended calibration procedure [15]. The sensor data were then streamed into MATLAB 2019b (MATLAB, MathWorks, Natick, MA, USA) via TCP/IP for analysis [16].

**Fig. 2:**
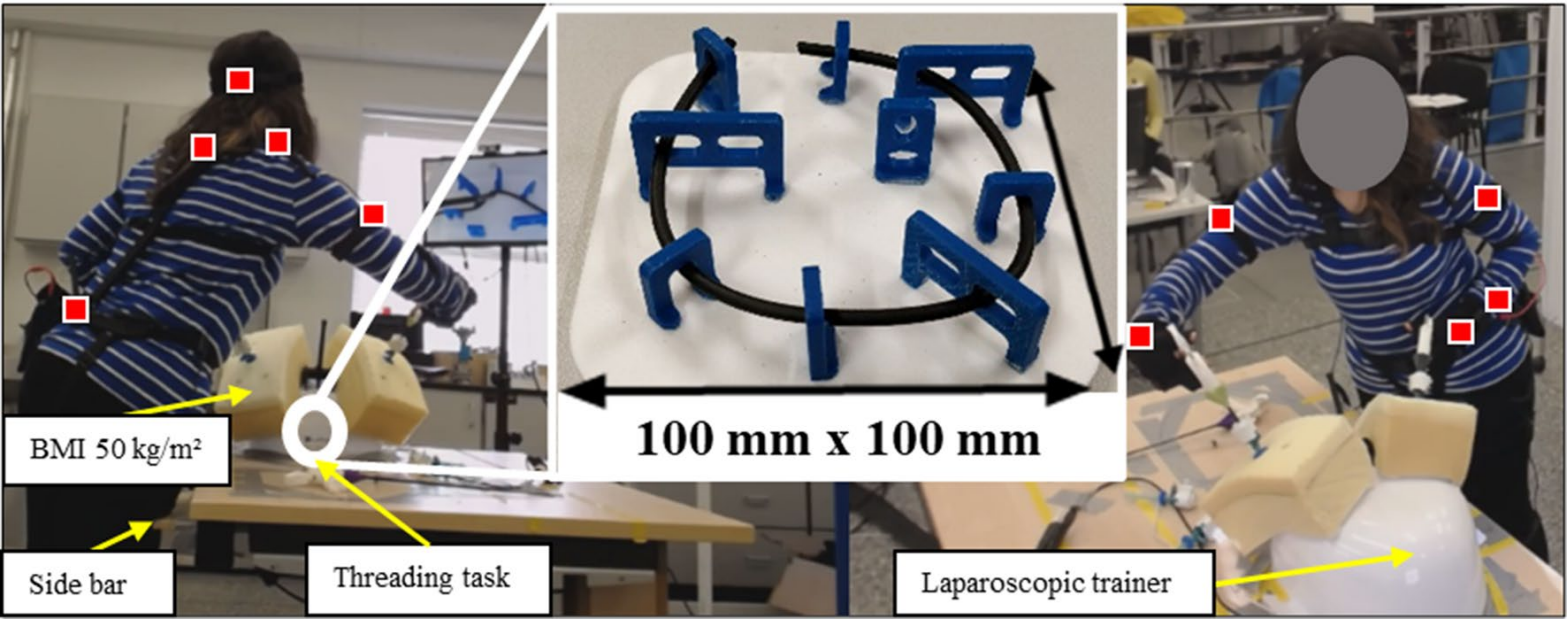
Experimental setup with BMI 50 kg/m^2^ analogue attached to laparoscopic trainer while completing a threading task. The visible IMUs in this figure have been highlighted with squares on the participant

Patient BMI was simulated using different thicknesses of foam [10]. The foam (*ρ* ≈ 37 kg/m^3^) was securely attached over the ports on the outside of the laparoscopic trainer using Velcro to ensure consistent implementation (Fig. 2). Foam with a thickness of 1.7 cm was used for the baseline BMI 20 kg/m^2^, 6.5 cm for 30 kg/m^2^, 9.5 cm for 40 kg/m^2^, and 11 cm for 50 kg/m^2^ [17]. The baseline model thickness was based on [18]. The foam thicknesses for all obese models were based on [19]. Moreover, for BMI levels ≥ 40 kg/m^2,^ a side bar of 7.5 cm in width was attached to the operating table, creating a wider table as used to accommodate patients with severe obesity in practice [20].

Participant anthropometrics were considered in the setup. An ergonomically optimal surface height has been established as 0.7–0.8 of a surgeon’s elbow height in neutral postures [21]. An acceptable surface height range of 84.6–107.8 cm was calculated for all surgeons based on their anthropometrics. Therefore, the operating surface height was set at 85 cm, which then ranged from ~ 86.7 cm for 20 kg/m^2^ to ~ 96 cm for 50 kg/m^2^ when including the BMI thicknesses to ensure optimized table ergonomics. The monitor height was also varied with participant height to ensure the monitor was approximately aligned with eye level [22].

### Testing procedure

The testing procedure consisted of performing a standard threading task typically used for laparoscopic training [23], when exposed to the different BMI levels. To complete the task, every participant was required to insert the thread into each of the outer frames until all eight outer frames were threaded (Fig. 2). A fixed laparoscope provided real-time visual feedback of the threading task on the monitor (Fig. 2).

The testing procedure required each participant to complete each BMI level twice. A maximum time threshold was not implemented during the experiments because fatigue is known to have an effect on kinematics and the subsequent dynamic workload [24]. Each participant was allowed familiarization trials with no BMI model present and the implementation order of the BMI levels was randomized to reduce learning bias [25]. Between each trial, participants were allowed a minimum of three minutes of rest to avoid pre-task-related fatigue.

Additional testing conditions were kept constant throughout the study including: the threading task distance from the ports within the trainer; port placement; laparoscope and its positioning and trocars. A contralateral port placement was selected as it has been shown to be a preferred setup among surgeons [26].

### Data analysis

The accelerometer data was filtered using a 4^th^ order Butterworth bandpass filter with a range of 0.2–20 Hz to remove the effects of gravity and noise. In addition, the gyroscope data was filtered using a 4^th^ order Butterworth lowpass filter with a cut-off frequency of 20 Hz to remove the effects of noise [14]. All sensors were defined in the local coordinate system, therefore, isolating the motion of each segment where a sensor was positioned. Moreover, the kinematic data for each segment was expressed relative to its parent segment further up the kinematic chain, following the same conventions as described in [17].

### Kinematic variables

To investigate the efficiency of motion, mean jerk, mean angular speed, and mean cumulative displacement (CD) were calculated for the upper body segments in Fig. 1. Mean CD also indicates the efficacy of motion when using 20 kg/m^2^ as the benchmark. Further, the total time taken to complete the task and CD were used as overall measures of dynamic workload.

Linear jerk |J| was computed by finding the magnitude of the first order differential of each component in the acceleration vector ($a_{x} , a_{y} ,a_{z}$) [11]. Angular speed $\left| \omega \right|$ was calculated by finding the magnitude of the angular velocity vector ($\omega_{x} , \omega_{y} ,\omega_{z}$). Displacement of upper body segments was calculated by double integrating the acceleration vector for each segment [12]. The magnitude of the displacement vector (${\text{s}}_{{\text{x}}} { },{\text{ s}}_{{\text{y}}} { },{\text{s}}_{{\text{z}}}$) for each segment was computed and summed to find CD [27].

### Subjective feedback

To understand whether the experimental setup and BMI models were realistic, a post testing questionnaire was included. The questionnaire comprised of five questions (Table 1). All questions were asked to the intermediate and expert surgeons (grouped as ‘experienced’); however, only questions 2 and 5 were asked to the novices because of their lack of previous experience. The questionnaire was answered once by each participant, after all trials had been completed.

**Table 1:** Post testing questions

Question number	Question	Results figure
1	How representative was the foam thickness of the intended BMI level?	Figure 4a
2	To what degree did the different foam condition impact the difficulty of the task?	Figure 4a
3	In terms of ‘movement feel’ when manipulating the instruments inside the laparoscopic trainer, please rate how representative the foam was of body fat?	Figure 4b
4	To what degree was the threading task representative of performing real surgery?	Figure 4b
5	To what degree did wearing the suit affect your movements during the trials?	Figure 4b

Participant responses were recorded on a 7-point Likert scale. A response of 6 indicated an exact representation/significant impact on movement/significant impact on difficulty, depending on the question, 3 represented ‘neutral’ and 0 indicated a completely unrepresentative setup/no impact on movement/no impact on difficulty, depending on the question.

### Statistical analysis

**Fig. 3:**
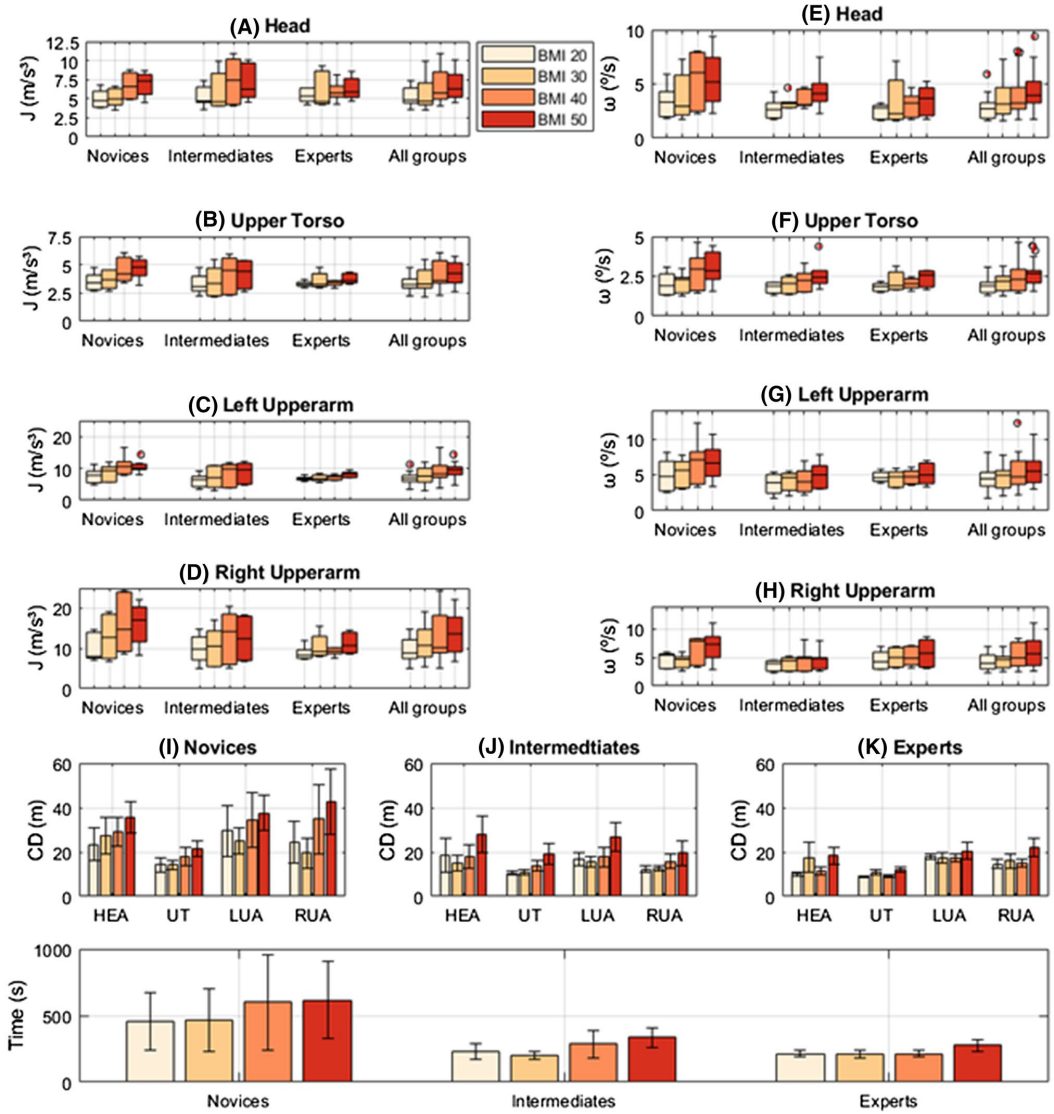
Mean jerk (**A–D**) and mean angular speed (**E**–**H**) boxplots for the defined segments of all experience groups displayed separately and together. In addition, mean (± standard error) cumulative displacement (Cu—Disp) of I) novices, J) intermediates and K) experts, for the following body segments HEA = head, UT = torso, LUA = left upper arm, RUA = right upper arm. Finally, L) mean (± standard error) task completion times for novices, intermediates, and experts. (Note: the boxplots in Fig. 3 are of different Y-axis magnitudes to suitably display the data)

Statistical analysis was completed in SPSS (IBM SPSS Statistics, v. 24, IBM Corp., Armonk, NY). The responses (Fig. 3) were calculated by grouping the participants into their respective experience groups and taking the mean of the trials per BMI. Due to the small intra-experience subsamples, normal distributions could not be assumed for parametric analysis; therefore, non-parametric analysis was performed by individually grouping all data by a single independent variable. When grouped by dependent samples (by BMI level), a Friedman’s two-way ANOVA was performed. When grouped by independent samples (by experience level), a Kruskal–Wallis test was performed. Both tests were performed for each anatomical area and kinematic variable. Where significant main effects were found, pairwise comparisons were carried out with Bonferroni correction. An alpha level of α ≤ 0.05 was set.

## Results

Each BMI level was completed twice by nine of the participants, while a single novice completed each level once due to lengthy completion times which prevented the possibility of test repeats. Results for the head, torso, and two upper arms (sensors 1, 2, 5 and 6) are presented due to the larger number of significant variances.

### Kinematics and workload results

Significant main effects of BMI level were seen for mean jerk, mean angular speed and cumulative displacement. Pairwise comparisons generally showed BMI 50 kg/m^2^ significantly increased the magnitudes of all variables compared to the baseline in all body segments (Table 2). Significant main effects of experience level were also found in all kinematic variables, with the most significant of these differences being for cumulative displacement, where novices displayed larger values than experts for the head and torso. Cumulative displacement magnitudes between the BMI level extremes (20 kg/m^2^ < 50 kg/m^2^) showed that 50 kg/m^2^ significantly increased the distance travelled by all segments. There was also a significant main effect of BMI on task completion time (*p* = 0.004), with pairwise comparisons showing that tasks took significantly longer during BMI 50 kg/m^2^ compared to 20 kg/m^2^ and 30 kg/m^2^.

**Table 2:** Statistical analysis

Body segments and kinematic parameters	Kruskal Wallis test (denoted by **K**) and pairwise experience group comparisons (denoted by e.g. **Exp/Int**/**Nov**)	Friedman’s test (denoted by **F**) and pairwise BMI comparisons (denoted by e.g. **20/50**)	
Head	Jerk (${\text{J}}$)	**K**: *p* = 0.998	**F**: *p* = 0.001, **20/40**: *p* = 0.032, **20/50**: *p* = 0.017, **30/40**: *p* = 0.039, **30/50**: *p* = 0.021
	Ang speed (${\upomega }$)	**K**: *p* = 0.088	**F**: *p* = 0.012, **20/50**: *p* = 0.017
	Cu–Disp (${\text{CD}}$)	**K**: *p* = 0.001, **Nov/Exp**: *p* = 0.001	**F**: *p* = 0.008, **20/50**: *p* = 0.007
Torso	Jerk (${\text{J}}$)	**K**: *p* = 0.176	**F**: *p* = 0.003, **20/40**: *p* = 0.047, **20/50**: *p* = 0.026
	Ang speed (${\upomega }$)	**K**: *p* = 0.279	**F**: *p* = 0.005, **20/50**:* p* = 0.003
	Cu–Disp (${\text{CD}}$)	**K**: *p* = 0.006, **Nov/Exp**: *p* = 0.004	**F**: *p* = 0.005, **20/50**: *p* = 0.004
Left upper arm	Jerk (${\text{J}}$)	**K**: *p* = 0.010, **Nov/Exp**: *p* = 0.011	**F**: *p* = 0.003, **20/40**:* p* = 0.021, **20/50**: *p* = 0.011
	Ang speed (${\upomega }$)	**K**: *p* = 0.028, **Nov/Int**: *p* = 0.024	**F**: *p* = 0.077
	Cu–Disp (${\text{CD}}$)	**K**: *p* = 0.086	**F**: *p* = 0.148
Right upper arm	Jerk (${\text{J}}$)	**K**: *p* = 0.075	**F**: *p* = 0.011, **20/50**: *p* = 0.039
	Ang speed (${\upomega }$)	**K**: *p* = 0.034, **Nov/Int**: *p* = 0.034	**F**: p = 0.006, **20/40**: *p* = 0.021
	Cu–Disp (${\text{CD}}$)	**K**: *p* = 0.081	**F**: *p* = 0.044, **30/50**: *p* = 0.05

Generally, BMI 50 kg/m^2^ caused upper body kinematics to significantly worsen compared to normal BMI models. Specifically, novices appeared to be markedly affected by BMI level once the BMI model reached 40 kg/m^2^ and intermediates were also noticeably affected in some cases, but not as extensively until BMI 50 kg/m^2^, whereas expert participants appeared affected only for BMI 50 kg/m^2^ with the lower BMI levels producing relatively consistent results. These results confirmed the hypotheses that significant deterioration in kinematics can be observed when surgeons are subjected to larger BMI models and that less experienced surgeons display worse kinematics.

### Questionnaire responses

**Fig. 4:**
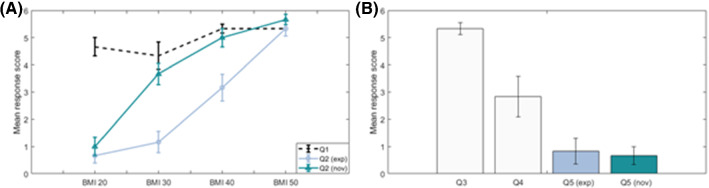
A. Mean (± Standard error) questionnaire responses for questions 1 and 2 (Table 1), B. Mean (± Standard error) questionnaire responses for questions 3,4 and 5 (Table 1). All questions were asked once per participant, at the end of all trials. Q2 and Q5 were asked to both experienced and novice participants

Post testing questionnaire responses indicated a positive response to the experimental methodology, supporting the ecological validity of the BMI models used, although less so the threading task protocol (Fig. 4).

## Discussion

The key purpose of this study was to objectively measure the effect of simulated obesity and severe obesity on the kinematics and dynamic workload of surgeons of varying experience levels. This research builds on previous work conducted into understanding the physical impact of MIS on the surgeon [9, 10, 17]. Mean jerk, angular speed and cumulative displacement magnitudes significantly increased in line with increasing model BMI for the head, torso, and both upper arms across all experience groups (Fig. 4, Table 2). Further, surgeon experience had a significant main effect on upper body kinematics, reinforcing previous findings that novices exhibited worse kinematics compared to more experienced surgeons [23].

The BMI 50 kg/m^2^ model caused significantly greater mean jerk magnitudes in all segments, indicating that all participants struggled to smoothly accelerate between changes in body position as compared to the baseline and obese model (Fig. 3A-D). Acceleration without jerk is a consequence of a static load or force such as gravity; therefore, jerk is felt as the increasing or decreasing change in force on the body [28]. A significant increase in jerk in conjunction with longer task completion times indicates the frequency and forcefulness of movements performed by the upper body increased, indicating less efficient performances that induced higher levels of required work to compensate for the larger model thickness and increased resistance against laparoscopic tool movement. Moreover, since degradation in motion control and smoothness were observed, and fatigue is a direct contributor to these [28], significant increases in jerk magnitudes indicate a more physically demanding task.

A similar set of statistical results were found for the angular speed of the head, torso, and dominant upper arm, where severely obese models caused significantly faster angular motion. Faster angular motion in conjunction with jerkier and more forceful movements suggests that these participant groups needed to repeatedly correct their upper arm movements to achieve the desired instrument manipulation [29]. The unexpected variability and skewed distribution in jerk and angular speed magnitudes within the expert group during 30 kg/m^2^ (Fig. 2), indicates that a single expert participant found this BMI level challenging kinematically, producing results analogous with the novices. This result is the product of a small sample size, and if a larger sample had been recruited these results would likely be outliers. In addition, these results also emphasize that even expert surgeons produce kinematics that could be optimised.

The BMI 50 kg/m^2^ model also had a significant negative effect on motion efficacy leading to larger cumulative displacement magnitudes (Fig. 3I–K). The effect of larger model circumferences at higher BMI levels caused accentuated motion altering optimal motion patterns represented by the baseline, as well as contributing to the variations in the other parameters. The significant increases in torso and upper arm cumulative displacement during BMI 50 kg/m^2^ compared to 20 kg/m^2^ indicate that these commonly reported at-risk areas struggled to adapt to the larger models compared to other areas of the upper body [7]. A larger magnitude for cumulative displacement is an increase in the total distance travelled; thus, a significantly larger dynamic workload was incurred at BMI 50 kg/m^2^ compared to the baseline and obese model.

The highly correlated results in all kinematic and workload-related variables indicate that BMI level had a considerable impact on a surgeon’s ability to perform to the optimal standard represented by the baseline. The thicker foam medium for the higher BMI models may have diminished the shoulder proprioceptive relationship with the trainer and led to the observed torso and upper arm behaviour [30]. All participants had previous experience of a laparoscopic training environment; however, this had not included BMI models (i.e. ≥ 30 kg/m^2^). Thus, any previous reinforced conscious or unconscious proprioceptive influence on the neuromuscular system during laparoscopic training may have naturally de-stabilized upper arm motion due to the unconventional high BMI training environment. Moreover, the effect of a thicker foam medium is likely to have restricted trocar-instrument movement supported by the perceived increase in task difficulty with increasing BMI (Fig. 4). This effect worked against instrument manipulation, induced more jerk (Fig. 3A–D), higher angular speeds (Fig. 3E–H), larger cumulative displacements (Fig. 3I–K), and longer task durations (Fig. 3L) as well as impairing the kinesthetic relationship between surgeon and laparoscopic trainer. Also, it could be argued that this is likely to be a replicated effect that is also seen in a clinical setting, caused by dense abdominal subcutaneous adiposity.

The key contribution and findings of this study are that performing procedures on patients with high BMIs (BMI > 40 kg/m^2^) creates a significantly more demanding task that induces higher levels of fatigue compared to normal BMIs (20 kg/m^2^) even for exercises of short duration. An increase in the levels of fatigue during physical tasks has been shown to increase the incidence of MSDs [7]. The results pertaining to the intermediate and expert groups offer the most clinical relevance within this study, as these groups have been regularly exposed to patients with a high BMI in surgery and are likely to have developed kinematic responses to manage such scenarios. However, these findings are concerning as MIS surgeons are exposed to patients with high BMIs with increasing regularity [10] and since many surgical procedures will have durations in excess of an hour, the cumulative impact of dynamic workload and fatigue with time may result in reduced surgical performance and worse patient outcomes. Investigation of surgeon ergonomics in the live surgery setting is needed to explore this impact further and develop interventions that can mitigate these effects. Trainee surgeons do need to gain experience in performing procedures on patients with a range of BMIs, but our study highlights that this group need training in ergonomic positioning rather than solely on improving their technical performance since suboptimal techniques could become ingrained and increase the risk of MSDs in their future careers.

The subjective feedback gathered from the participants regarding the experimental setup and BMI models provided a largely positive response to the conducted methodology. The methodological strengths include the realism of the BMI models in terms of movement feel and thickness, as well as the objective instrumentation system having a negligible effect on surgeon kinematics. The threading task was reported to be the most challenging; such tasks are primed for specific surgical skill development rather than surgery itself; however, other standardized tasks or simulated procedures should be considered in the future testing.

There are limitations that should be acknowledged when interpreting the results from this study. The relatively small heterogenous cohort of surgeons and completion of one simulated experimental task could reduce the generalizability of the relationships between BMI, experience level and upper body kinematics. The use of foam as the material to simulate adipose tissue does not fully replicate the material properties of adipose tissue. Further, the models also did not account for the differences in adipose tissue distribution (visceral/subcutaneous), nor how increased intra-abdominal adiposity effected surgeon kinematics.

However, the subjective feedback gathered largely supports foam as an appropriate representation. The subjective feedback on each BMI level was gathered post experiments rather than after each BMI level. Additionally, the contralateral port placement may not be routinely used by some surgeons who prefer to vary port sites depending on patient BMI. Nevertheless, the setup was implemented through the recommendation from practicing gynaecologists [26]. This study considered only upper body kinematics, the effect of patients with obesity on the wider biomechanical impact of surgeons is also of relevance to surgeon wellbeing. Finally, despite the mixed gender of the participant cohort, the impact of patient BMI on male/female surgeons was not investigated, which is an aspect of bariatric LS that should be considered in the future studies.

## Conclusion

Performing LS on high (40 and 50 kg/m^2^) BMI models is physically more demanding compared to performing on 20 and 30 kg/m^2^ models. More specifically, high BMI models degraded the efficiency and efficacy of surgeon head, torso and upper arm movement and increased their dynamic workload. Novice surgeons need exposure to high BMI models early in their career, to minimize the increase in workload through the optimization of kinematic performance while still in training. Furthermore, with increasing worldwide obesity levels, additional ergonomic training and support should be considered for surgeons who perform LS since this may reduce their risk of future MSDs.

## Data Availability

The code written to perform this analysis can be found: 10.24433/CO.7129140.v1
